# Introduction

**DOI:** 10.1097/pq9.0000000000000075

**Published:** 2018-04-17

**Authors:** Matthew Niedner

## Proceedings of the Improved Pediatric Sepsis Outcomes Colloquium-Dallas TX, December 2017

The introductory paragraph of anything relating to pediatric sepsis has a familiar refrain about it. It goes something like… Pediatric sepsis is common, is responsible for significant morbidity and mortality, and is the leading cause of pediatric deaths worldwide—striking down both healthy and at-risk children.^1–4^ There is a large evidence-based therapeutic arsenal available to treat sepsis and reverse shock, with contemporary consensus guidelines—and ample data to show that increased adherence to such guidelines significantly improves outcomes, including survivorship.^5–9^ Pediatric sepsis is a multi-billion dollar problem annually in the United States—with costs that rise year after year.^10^ Not surprisingly, health systems are experiencing increased levels of mandatory performance and outcome reporting from national and state regulatory bodies and looming pay-for-performance metrics.^7^

Much of the clinically relevant scientific literature in pediatric sepsis remains descriptive. Clinicians caring for children with sepsis do not get to choose the case mix of their patients, including risk factors, underlying conditions, and pathogens that infect the children receiving their care. But these clinicians do have meaningful influence over many of the processes of care delivery—such as monitoring standards, prompt recognition, antibiotic selection, time to shock reversal, team organization, and order sets, to name a few. And there exists great inter-individual and inter-institutional variability in many of these important processes, presenting extensive ground for improvement science to gain traction.

The American College of Critical Care Medicine recently published the 2014 update to its Clinical Practice Parameters for Hemodynamic Support of Pediatric and Neonatal Septic Shock.^5^ While much of the “what to do and when to do it” embedded in the resuscitation and stabilization bundles was largely unchanged, there were 2 very substantive, structural additions. On the front end, a recognition bundle to systematically screen for sepsis using a standardized trigger tool was added. And on the back end, recommendations were made to implement a performance bundle to measure adherence to key and time-sensitive care processes.

The most pressing work at hand is to take what we already know works in patients with sepsis and ensure those things are happening as reliably and efficiently as possible. It has never been very realistic to simply believe care delivery is good and reliable because our intentions are good and we are sharp-minded—we need to do due diligence. On an Earth Day in a 1971 comic strip, Pogo said, “We have met the enemy, and he is us.” To achieve the biggest, fastest gains in sepsis-related outcomes, we need to improve our systems of care delivery. We need to speed up and improve our detection of sepsis and we need to treat it quickly and correctly when it occurs.

To this end, The Children’s Hospital Association launched the Improving Pediatric Sepsis Outcomes Collaborative in 2016—a multicenter quality improvement network to accelerate and disseminate improvements in the care of children with sepsis. More than 4 dozen children’s hospitals have banded together to tackle this challenge. At the most recent semiannual Improving Pediatric Sepsis Outcomes Colloquium in Dallas, Texas in December of 2017, more than half of participating sites submitted abstracts which were presented in poster or oral format. Although unified under a shared goal, the work presented is diverse in scope and methods. Read on to learn:

- how organizational strategies have improved physician engagement;
- how sites have significantly improved the accuracy and timeliness of sepsis identification;
- how detection tools have been tailored to specific care settings;
- how local health record systems have been leveraged successfully;
- how trigger tools have had sensitivity and specificity optimized to achieve favorable receiver-operator characteristic curves;
- how time to key interventions, like fluids and antibiotics, have been slashed;
- how safer intensive care transfers have been achieved;
- how severity-adjusted sepsis mortality has been reduced.

The following 18 abstracts from this venue represent some of the great work going on in the ISPO collaborative. Here, we proudly share insights from our colleagues in the trenches battling pediatric sepsis with quality improvement strategies.

## References

[1] Hartman ME Linde-Zwirble WT Angus DC Trends in the epidemiology of pediatric severe sepsis. Pediatr Crit Care Med. 2013;14:686 693. 23897242 10.1097/PCC.0b013e3182917fad

[2] Wang H Dwyer-Lindgren L Lofgren KT Age-specific and sex-specific mortality in 187 countries, 1970-2010: a systematic analysis for the Global Burden of Disease Study 2010. Lancet. 2012;380:2071 2094. 23245603 10.1016/S0140-6736(12)61719-X

[3] Weiss SL Balamuth F Hensley J The epidemiology of hospital death following pediatric severe sepsis: when, why, and how children with sepsis die. Pediatr Crit Care Med. 2017;18:823 830. 28549024 10.1097/PCC.0000000000001222 PMC5581233

[4] Cvetkovic M Lutman D Ramnarayan P Timing of death in children referred for intensive care with severe sepsis: implications for interventional studies. Pediatr Crit Care Med. 2015;16:410 417. 25739013 10.1097/PCC.0000000000000385

[5] Davis AL Carcillo JA Aneja RK American College of Critical Care Medicine clinical practice parameters for hemodynamic support of pediatric and neonatal septic shock. Crit Care Med. 2017;45:1061 1093. 28509730 10.1097/CCM.0000000000002425

[6] Dellinger RP Levy MM Rhodes A; Surviving Sepsis Campaign Guidelines Committee including the Pediatric Subgroup. Surviving sepsis campaign: international guidelines for management of severe sepsis and septic shock: 2012. Crit Care Med. 2013;41:580 637. 23353941 10.1097/CCM.0b013e31827e83af

[7] Hershey TB Kahn JM State sepsis mandates—a new era for regulation of hospital quality. N Engl J Med. 2017;376:2311 2313. 28528558 10.1056/NEJMp1611928

[8] Seymour CW Gesten F Prescott HC Time to treatment and mortality during mandated emergency care for sepsis. N Engl J Med. 2017;376:2235 2244. 28528569 10.1056/NEJMoa1703058 PMC5538258

[9] Han YY Carcillo JA Dragotta MA Early reversal of pediatric-neonatal septic shock by community physicians is associated with improved outcome. Pediatrics. 2003;112:793 799. 14523168 10.1542/peds.112.4.793

[10] Torio CM Moore BJ National inpatient hospital costs: the most expensive conditions by payer, 2013, statistical brief #204. 2016 Healthcare Cost and Utilization Project (HCUP). 27359025

